# Laboratory Evaluation of ESGFIBER in Asphalt Paving Mixture

**DOI:** 10.3390/ma15165754

**Published:** 2022-08-20

**Authors:** Agathon Mrema, Hyeong-Su Kim, Jae-Kyu Lim, Jae-Jun Lee

**Affiliations:** 1 Department of Civil Engineering, Jeonbuk National University, Jeonju-si 54896, Korea; 2 ESG Industry Company Limited, Daejeon 34328, Korea; 3 Korea Institute of Civil Engineering and Building Technology, Goyang-si 10223, Korea

**Keywords:** ESGFIBER, asphalt concrete, indirect tensile strength, deformation test, Marshall mix design, tensile strength ratio, Hamburg wheel tracking test

## Abstract

The global desire to improve the performance of road pavements and move towards a sustainable transportation system has immensely encouraged the usage of fibers in asphalt paving materials. In this study, glass fibers trademarked as ESGFIBER produced by the ESG Industry company Limited from Daejeon, Korea were added in dense-graded asphalt mix. The purpose of this study was to evaluate effects that fibers have on volumetric properties, mechanical properties, and long-term performance of asphalt concrete mixes. ESGFIBER were mixed together with aggregates and asphalt binder in asphalt mix and five different asphalt mixes with different dosage of fibers were evaluated in this study. The Marshall mix design method was used for designing all asphalt mixes, and laboratory tests indirect tensile strength test, deformation strength test and Hamburg wheel tracking test were conducted to evaluate moisture susceptibility, fatigue cracking behavior and rutting resistance of asphalt concrete mixes. The results showed that when ESGFIBER were added in asphalt mix moisture susceptibility, fatigue cracking and rutting resistance were both improved. The usage of ESGFIBER in asphalt concrete mixes can be very beneficial since the mechanical and long-term performance were improved upon the addition of fibers.

## 1. Introduction

The global desire to improve the performance of road pavements and move towards a sustainable transportation system has immensely encouraged the usage of fibers and other additives in paving materials. Additives have been added in both flexible (asphalt concrete) and rigid (cement concrete) pavements for the purpose of improving durability, mechanical properties, reduce costs and lessen environmental pollutions [1,2,3,4,5]. Fibers together with polymers are the most used additives in flexible pavements, and many types of fibers have been applied in road pavements saving different purposes. The general idea of adding or reinforcing fibers into road pavement mixtures is, fibers are added to provide and/or improving some desired properties which are lacking in the certain type of a pavement mixture and hence enhance the overall performance of pavement [6,7]. For asphalt pavements, fibers are added into asphalt concrete (AC) mixes mainly to add more strength to the mixture for a dense-graded asphalt mixtures and reduce asphalt drain down for gap and open-graded asphalt mixtures. However, there is an increase of number of studies where fibers were used for other purposes, such as reducing noises and improving the smoothness and self-healing of cracks [1,8,9,10].

### 1.1. Background

The usage of fibers together with other construction materials can be dated back to 4000 years ago, when an old arch in China was constructed with clay soil mixed with fibers [11]. The early published work of fibers used in asphalt pavement goes back to the 1920s, where in the USA asbestos fibers were used in the asphalt pavements and improved its performance. Asbestos fibers continued to be used until in the 1960s when health and environmental concerns put an end to it [12,13]. In the 1930s, cotton fibers were commonly used in asphalt pavements but they suffered from the problem of degrading over time [14]. In the 1950s, Zube [15] used wire mesh which was placed under an overlay in an attempt to prevent reflection cracking. Since then, the usage of fibers in asphalt pavement has been increasing substantially with more different types of fibers being used [16,17].

During the early applications of fibers, it is known that fibers were mainly used in dense-graded asphalt mixtures for the purpose of adding strength to the mixture [1]. The introduction of the stone matrix asphalt (SMA) in the 1960s led to an increase in the usage of other types of fibers which have greater absorption capabilities rather than strength. SMA was getting very popular because it was found to be resistant to wear and tear of tires, highly resistant to permanent deformation and more durable than the convectional dense-graded asphalt mixtures [17,18]. However, SMA suffered from the problem of having a high amount of asphalt binder and more coarse aggregate which led to binder drain down. As a result, SMA mixtures needed proper type and amount of some stabilizing additives in order to retain the binder during a production and placement. Fibers with high absorption capabilities, such as mineral, rockwool, and cellulose fibers, were used in SMA mixtures and they were found to be very helpful in stabilizing or preventing asphalt binder drain down [19,20].

The state of asphalt pavement’s durability and performance can be compromised by the occurrence of distresses. All pavement types are subjected to variety of distresses, however the development of these distresses need not to be viewed with alarm unless the distresses occur early in the design life of a pavement [21]. The common types of distresses that occur in asphalt pavement are cracking, rutting, shoving, potholes, bleeding, and skidding. The distresses in asphalt pavements are mostly caused by climatic conditions and the traffic loads [21,22,23,24]. Since it is impossible to control the climate conditions and sometimes hard to control traffic loadings, fibers are more often added in asphalt mixtures to delay the occurrence of distresses. Fibers with high tensile strength such as glass fibers, polyester, basalt fibers, aramid and polypropylene are highly resistant to cracking, rutting and potholes [25,26,27,28,29,30,31].

ESGFIBER were used in this research to evaluate the influence of fibers in dense-graded AC mixes. ESGFIBER, a type of glass fiber developed by the ESG Industry in Daejeon City, Korea for a purpose of being used on road pavements. Glass fibers are now widely used in hot mix asphalt (HMA) since they have been proven to be beneficial due to their excellent mechanical properties [7,32]. Glass fibers have high strength, high stiffness, elongation of about 3–5%, and they are resistant to oxidation and moisture [33,34,35,36]. Glass fibers in AC mixes can increase stiffness, tensile strength and improve the resistance to fatigue cracking and rutting. Moreover, Khangali and Tortum [37] studied the fracture characteristics and conditions of glass fibers in HMA and their results showed that glass fibers had a great impact on fracture energy, where fracture energy increased with the increasing of glass fiber content and fracture angle. In other research, it was also found that glass fibers improved substantially the rutting behavior, fracture resistance and crack intensity factor of HMA mix [37,38,39,40]. The overall results suggested that glass fibers are ideal for the improvement of the asphalt pavement performance.

### 1.2. Objective and Scope

The objective of this study was to evaluate the effects of adding ESGFIBER in dense-graded AC mixes. Laboratory investigations were conducted to evaluate the volumetric and mechanical properties, and long-term performance of AC mixes with different dosage of ESGFIBER. In this study there were 5 different mixes with 0%, 0.2%, 0.3%, 0.4% and 0.5% of fiber content measured according to the weight of aggregate. These mixes were named as control mix, mix 1, mix 2, mix 3, and mix 4 respectively. The laboratory tests adopted in this study included the Marshall mix design, indirect tensile strength test, Hamburg wheel tracking test, and deformation strength test. The results obtained were analyzed and compared for each test conducted and the optimum fiber content. The one with best overall results was acknowledged.

## 2. Materials and Methodology

### 2.1. Materials

**Figure 1:**
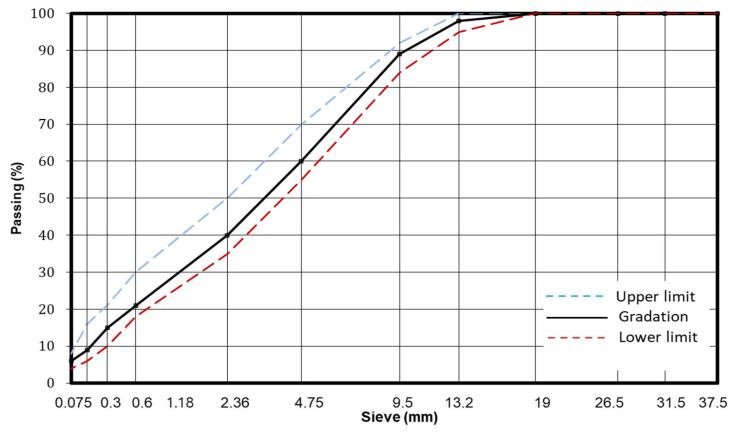
Aggregate gradation.

Asphalt binder known as AP5 Premium was chosen to be used in this study for all mixes. AP5 Premium was obtained from S-Oil Corporation Industry Seoul, Korea, and its properties are given in Table 1 below. Coarse and fine aggregate used in this study were obtained from AR Concrete company in Jeollabuk-do city, Korea. Aggregate gradation used had the nominal maximum aggregate size of 13 mm and its gradation, upper and lower limits are given in Figure 1 below.

**Figure 2:**
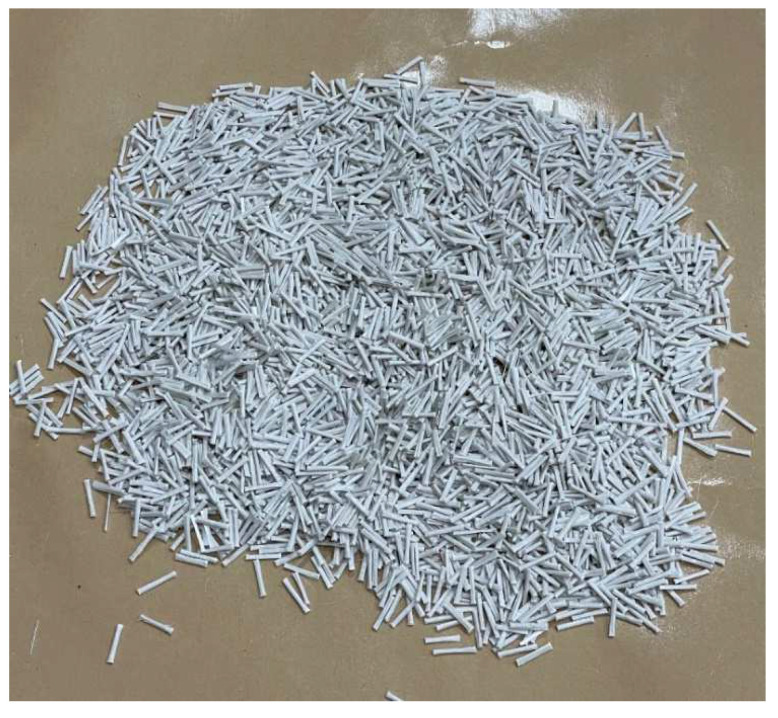
Physical appearance of ESGFIBER.

ESGFIBER used in this study were provided by ESG Industry Company Limited in Daejeon, Korea. The normal appearance of ESGFIBER is as shown in Figure 2 and its properties are given in the Table 2 below.

### 2.2. Specimen Preparation

In this study, ESGFIBER were incorporated with aggregate and asphalt binder in dense-graded AC mix. There were 4 different mixes with 0.2%, 0.3%, 0.4% and 0.5% by weight of aggregate and a control mix the one without fibers. For mixes with fibers, fibers were first mixed with aggregate for about 60 s to ensure fibers were well dispersed in aggregate. Subsequently, heated asphalt binder was added into a mixture of aggregate and fibers. This method is known as the dry mixing method and it is mostly preferably for AC mixes with fibers [42,43]. After mixing the hot asphalt mixture, the mixture was heated again for about an hour before it was placed into a mold, compacted, and cured for 24 h prior to testing.

### 2.3. Marshall Mix Design Test

The HMA mixture needs to be well designed so that it can fulfill the requirements required for structural and pavement surface characteristics and withstand heavy traffic loads under different climatic conditions. The Marshall mix design method was used in designing AC mixes used in this research. The volumetric properties, Marshall stability, and Marshall flow of all mixes were obtained through this method. The Marshall mix design was followed in accordance with Korean standards for the construction sector KS F 2337 [41]. In this study, there were 5 different dense-graded AC mixes and Marshall mix design test was conducted for each mix. For each mix, three different asphalt binder contents were tested for volumetric and strength criteria to select the optimum asphalt binder content (OAC). The volumetric characteristics and strength properties of AC mixes with same asphalt binder content were carefully studied to investigate the effects of adding different contents ESGFIBER. The OAC for all mixes were chosen considering that both volumetric and strength criteria met the required standards.

### 2.4. Indirect Tensile Strength Test (IDT)

The indirect tensile strength test (IDT) is used to evaluate tensile properties of AC mixes in terms of tensile strength, strain (displacement) and toughness. The repeated heavy traffic loadings over a long period could affect the strength of an asphalt pavement causing fatigue cracks. IDT test is commonly used to determine indirectly the tensile strength of asphalt mixture against the formation of fatigue cracks. In this study, the IDT test was conducted in accordance with Korean Standards KS F 2382 [41], where a cylindrical specimen with weight of 1150 g and diameter 101.6 mm compacted 75 times on both sides was loaded with a compressive load at a constant rate of 50 mm/min acting parallel to and along the diametric plane of a cylindrical specimen. During IDT testing, toughness of AC mixes was also calculated. The toughness of AC mixtures, also known as stiffness, is a mechanical property representing the ability of AC to resist fractures after the initial crack [44] and it is calculated from the total energy dissipated during specimen fracturing process.

Additionally, the indirect tensile strength ratio (TSR) test was conducted to evaluate the moisture susceptibility of AC mixes. The availability of moisture in asphalt pavement is considered to be the root cause of many serious pavement damages such as stripping of aggregate, emulsification of the asphalt and freezing of entrapped water inside the pavement [45,46,47,48]. During testing, TSR values were obtained from the average IDT of wet conditioned specimens (soaked in water bath at a temperature of 60 °C for 24 h) by average IDT of dry unconditioned specimens. Each set of fiber content had six (6) specimens, three conditioned and three unconditioned specimens with air voids of 7 ± 0.5% and TSR value of 80% or above was considered acceptable. The TSR test procedures were conducted in accordance to Korean Standards KS F 2398 [41].

### 2.5. Hamburg Wheel Tracking Test (HWT)

Hamburg Wheel Tracking test (HWT) is a laboratory test normally used to evaluate moisture sensitivity and rutting resistance of the AC mixture. The HWT has two modes dry condition mode and submerged (wet) condition mode, in this research only submerged mode was applied. Although the HWT wet condition mode is usually used in AC mix design to evaluate the long-term performance of both rutting and stripping susceptibility, many researchers consider this mode to be an indicator of stripping susceptibility rather than rutting susceptibility [49]. During experimentation, specimens were submerged in hot water bath at a temperature of 50 °C. Then, a steel wheel with an applied load of 705 N was tracked back and forth to induce rutting and stripping of the aggregate. The test was performed in accordance with AASHTO T 324 standard test procedure and specimens were compacted to 7% air voids.

### 2.6. Deformation Strength Test

The deformation strength test was another test in this study used to evaluate the rutting resistance of AC mixtures. This test was recently developed in Korea as an alternative to Marshall Stability test, which is believed to be outdated. The deformation strength test adopted the application of a load to the limited area on the specimen rather than applying a load to the whole specimen cross-sectional area so as to simulate the circular spot which suffers the stresses when a wheel is in contact with the road. The round edge loading head was used to create a concave form of deformation on AC specimen, which is considered as similar to the contact area as tire loading [50,51]. Cylindrical specimens similar to Marshall specimens were submerged in a water at 60 °C for 30 min, then loaded at a speed of 30 mm/min using Marshall loading machine. The resistance to the formation of a dimple on a specimen is what is known as deformation strength and it is obtained from the following equation.

$$S_{D}=\frac{0.32P}{{\left[10+\sqrt{20y-y^{2}}\right]}^{2}}$$

where *S_D_* = deformation strength; *P* = maximum load; *y* = deformation.

## 3. Result

### 3.1. Marshall Mix Design Test

The results of Marshall mix design test are categorized into two categories, volumetric properties of the compacted AC mixture and strength properties (Marshall stability and Marshall flow) results. The volumetric properties of compacted AC mixture include optimum asphalt binder content (OAC), air voids percentage (Va), voids in the mineral aggregate (VMA), and voids filled asphalt binder (VFA). The results of volumetric analysis are given in the Table 3 below. The OAC percentage was chosen at 4% air voids, and results of OAC for AC mixes with different ESGFIBER contents decreased with the increase of fiber contents. This means that the addition of fiber led to the decreases of the amount of asphalt binder required to reach the 4% air voids, while meeting the other mix designing criteria. In many previous cases, more asphalt binder was needed to accommodate the inclusion of fibers in AC. However, the addition of ESGFIBER in AC mix led to a decrease of binder. Normally, fibers have a tendency of absorbing asphalt binder which leads to the increase of AOC, but ESGFIBERS seemed to have less or none absorption capabilities which led to a decrease of asphalt binders.

Moreover, the air voids percentage for asphalt binder content decreased with the increase of the ESG fibers as shown in Table 3 below. For the theoretical maximum specific gravity (G_mm_) and bulk specific gravity (G_mb_) of the AC mixtures, the values slightly increased for mixes with fibers as a result of reduction of asphalt binder weight, which led to the increase weight of aggregate and fibers which have higher actual density than asphalt binder. The results of VMA and VFA of control mixture and mixes with fiber for a chosen OAC are also given in Table 3 below. The VMA and VFA values decreased for mixes with fibers compared to the control mix. For VMA, the inclusion of ESGFIBER led to the reduction of volume of void space between aggregate particles through a decrease of effective asphalt content which was caused by a decrease of OAC for mixes with fibers. The same applied to a decrease of VFA values, a decrease of effective asphalt content led to a decrease of volume filled with asphalt (VFA).

**Figure 3:**
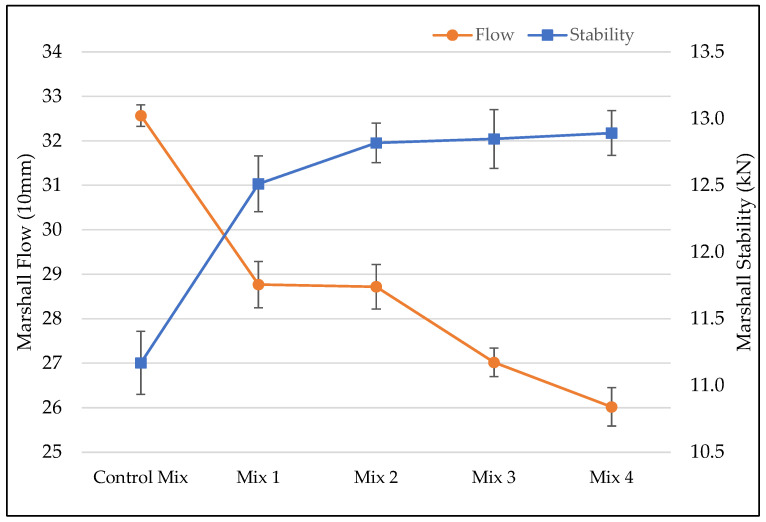
Marshal Stability and Flow results.

The Marshall stability results for asphalt binder contents used in Marshall mix design test are given in the Table 3 below. For each asphalt binder content, the stability values increased upon the addition of ESG fibers. However, volumetric properties of mixes with different fiber contents but same asphalt binder content were very different and some failed to meet the required standards in a mix design. This led to not keeping the same asphalt binder content for all mixes but rather using the OAC of each fiber content. Marshall stability and Marshall flow results for AC mixes at their OAC are given in the Figure 3 below. For Marshall stability, the values increased with the addition of fiber. Marshall stability increased with the increase of the ESGFIBER in AC mix and, a mix 4 with 0.5% of fiber content had the highest stability of all mixes. Marshall stability testing provides important information about the strength of AC mixes at high temperature which can be correlated with rutting resistance. AC mixes with high stability are considered to also have high rutting resistance. Marshall flow for AC mixes with ESGFIBER decreased compared to the control mixture. Marshall flow is a measure of the deformation in terms of elasticity and plasticity of the compacted AC specimen. For AC mixes with high flow value, they are considered to be more plastic, and the ones with low flow value are considered to be brittle. Although all the flow values obtained in this research were within the required limits, the decrease of flow value was mainly attributed to the reduction of asphalt binder for mixes with fibers.

### 3.2. Indirect Tensile Strength Test (IDT)

**Figure 4:**
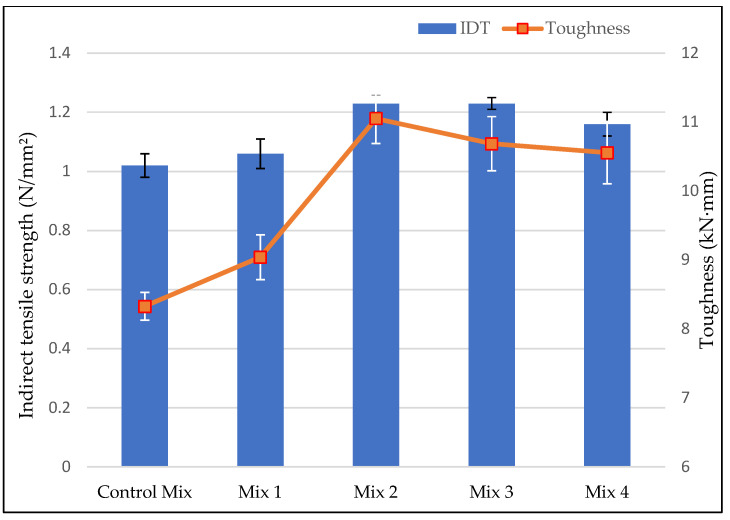
Indirect Tensile Strength and Toughness results.

The IDT test results showed that AC mixes with fibers had better fatigue cracking resistance performance than the control mix. The IDT values increased and reached its peak at mix 2 and mix 3 (0.3% and 0.4% of fiber content) and decreased for AC mix with highest fiber content which in this study was mix 4 as shown in Figure 4. The increase of IDT was observed even after the reduction of the asphalt binder for the mixes with fibers. Although it can be suggested that the increase of aggregates after the reduction of asphalt binder caused AC mix to be stiffer and stronger, a decrease of IDT value at mix 4 gave more insight on a contribution of ESGFIBER in the increase of IDT values. The peak IDT value at mix 2 and mix 3 was 20% increase from the control mix. Toughness of the AC also increased for mixes with ESGFIBER. Mixes with higher toughness values have more stiffness to resist the fracture after the initial the cracks.

**Figure 5:**
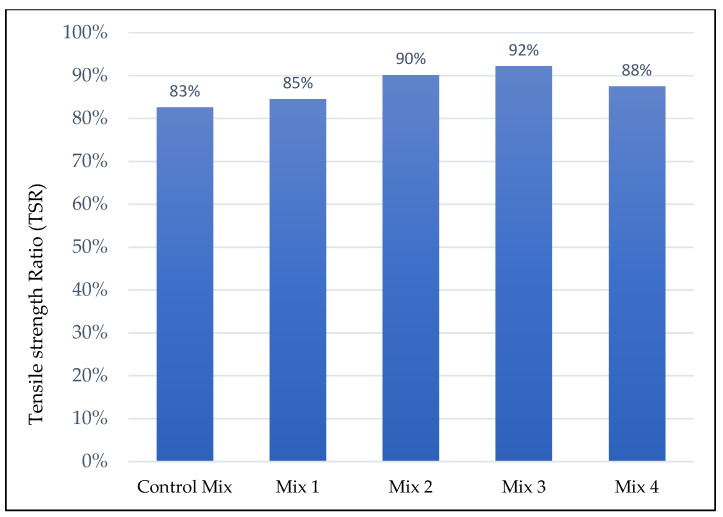
Indirect Tensile Strength Ratio results.

Toughness is an important parameter in AC mixes because, cracks at some point during a pavement service life are going to occur and the capability of AC mix to slow down the propagation of cracks determines the length of service life of a pavement under heavy loadings. Toughness reached its peak at mix 2 with 0.3% of fiber content and started to decrease for mix 3 and mix 4, but it was also observed that even a decrease for higher fiber contents the toughness was higher than a control mix. Fibers firmly held the aggregate particles inside the matrix and prevented them from moving, which made the mixes stiffer, hence leading to higher IDT and toughness results for mixes with fibers. Moreover, the TSR results given in Figure 5 below shows an increasing trend for mixes with fibers. The addition of fibers improved the moisture resistance of AC mix, and mix 3 had the best TSR results overall.

### 3.3. Hamburg Wheel Tracking Test (HWT)

**Figure 6:**
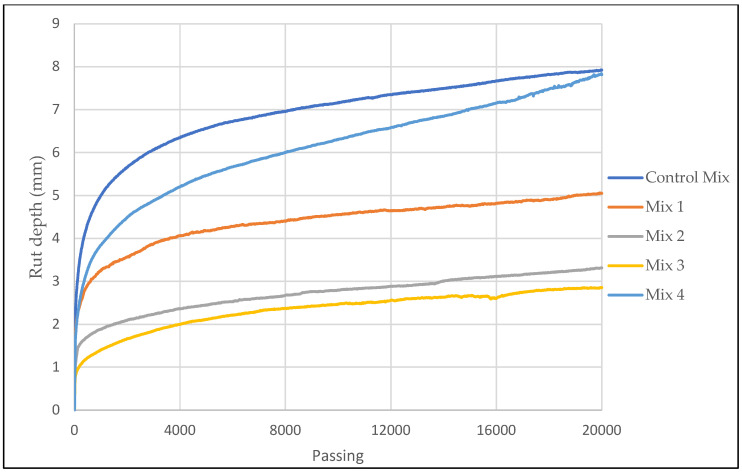
Hamburg Wheel Tracking test results.

**Figure 7:**
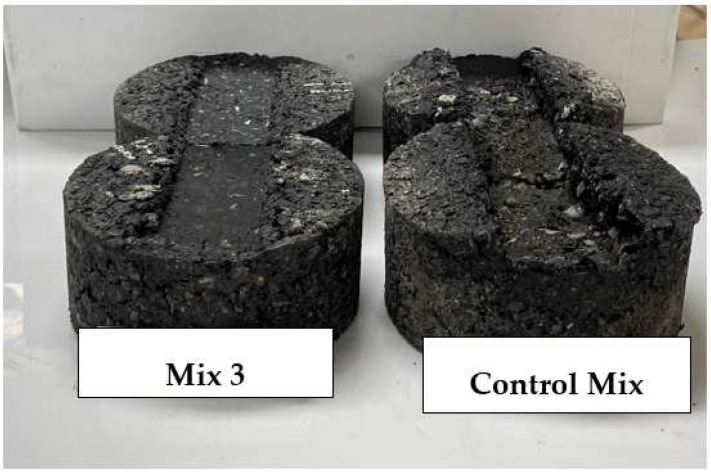
Specimens after Hamburg wheel tracking testing.

The rutting and stripping performance of asphalt pavement are important factors to be considered in asphalt pavement design. The increase of heavy traffic loadings and availability of moisture in asphalt pavements can shorten the life span of a pavement. HWT test was taken for laboratory evaluation of rutting and stripping susceptibility, and results are as shown in Figure 6 below. AC mixes with fibers had significantly low rut depth compared to a control mix. The inclusion of ESGFIBER in the AC and reduction of binder as the result of addition of fibers made the AC mix to become more stiffer and therefore more resistant to rutting. Moreover, structurally, fibers provide extra bonding interface between aggregates and binder which can transfer more stress and hence increase rutting resistance. Both mix 2 and mix 3 with 0.3% and 0.4% of fiber content performed better than other mixes, in terms of rutting resistance but at some point, near the end of experiment it showed a start of significant moisture damage in the mix as it has shown in Figure 6 below. Although both mixes had a good TSR results, in the long run, under constant wheel loading, it started to be damaged by the moisture. Other mixtures at some point also had this problem, which can be explained as being mostly caused by the designed AC mix rather than the performance of fibers. Although all mixes showed stripping potential, the rut depth of all mixes met the required standards, which is less than 12 mm rut depth after 20,000 passes. Moreover, control mix and mix 4 with 0.5% of fiber content had nearly the same rutting depth after 20,000 passes. Figure 7 shows control mix and mix 3 specimens after the experiment. The control mix specimen appeared to be very damaged by rutting and moisture compared to mix 3 specimen.

### 3.4. Deformation Strength Test

**Figure 8:**
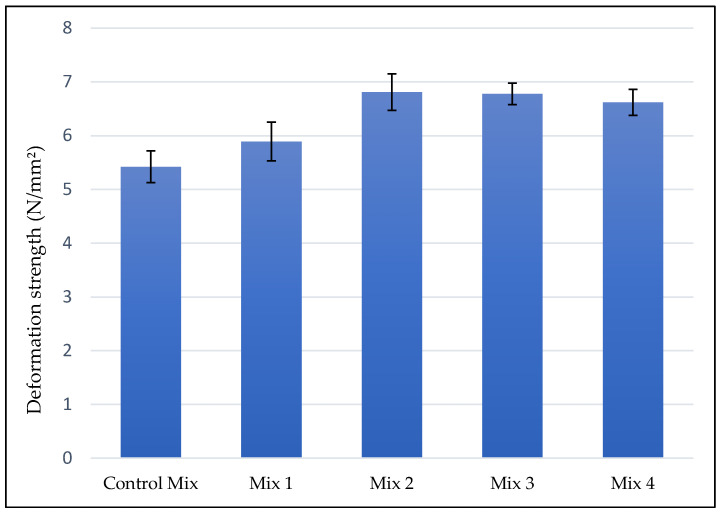
Deformation Strength test results.

The results of deformation strength test for mixes with different dosage of fiber are given in Figure 8 below. Results showed that fibers improved the high-temperature rutting resistance of AC mix. The amount of fiber content in the AC mix influenced the increase of *S_D_* values significantly, *S_D_* steadily increased as the amount of fiber content increased and reached its peak at mix 2 which had 0.3% of fiber content. At mix 2 *S_D_* value increased by 26% compared to control mix. As shown in Figure 8 below, for mix 3 and mix 4 which had 0.4% and 0.5% of fiber contents according to weight of aggregate, the *S_D_* value decreased from that of mix 2, which indicates that for a higher amount of fiber, the rutting resistance performance of AC starts to drop. The increase of rutting resistance for mixes with ESG was due to the good distribution of fibers in asphalt matrix which highly resisted the shear displacement and firmly prevented aggregate particles from any movement.

## 4. Conclusions

This study was conducted to evaluate the performance of ESGFIBER in dense-graded AC mix. The laboratory tests were taken to evaluate fatigue cracking, rutting resistance, toughness, and moisture sensitivity of mixes with fibers and a control mix, the one without fibers. The following conclusions were reached based on the laboratory investigations:

Mechanical properties and performance of AC mix were improved after the addition of ESGFIBER. Fatigue cracking resistance, toughness, moisture susceptibility, and rutting resistance were all enhanced upon the addition of fibers.

After the analysis of the results obtained from all mixes, mix 2 which had 0.3% of fiber content according to the weight of aggregate demonstrated the best overall performance over the other mixes in this study.

The addition of ESGFIBER led to a reduction of asphalt binder in order to achieve the volumetric characteristics required for dense-graded mixes. Although the reduction of a binder can be seen as advantageous since binder is the more expensive than the rest of materials in AC mix, further investigations regarding the durability of AC mixes with ESGFIBER need to be conducted.

The volumetric characteristics and Marshall flow for mixes with ESGFIBER need further investigation to understand the effects and mechanism of ESGFIBER in a compacted AC. The variation in results in VFA, VMA, and flow for mixes with different ESG fiber contents raised concern regarding their cause and whether they have any long-term effects on the performance of AC pavement.

Lastly, although the ESGFIBER performed well in AC mix according to laboratory investigations, the nature of AC mix being complex heterogenous composite material makes it difficult to control the variability of laboratory tests and field performance due to complex working mechanisms and environmental interferences. Field evaluations should be conducted to verify the performance of ESGFIBER in real road pavements.

## Figures and Tables

**Table 1:** Properties of AP5-Premium asphalt binder.

Property	Test Value	Standards
Penetration (25 °C, 100 g, 5 s) 0.1 mm	63	KS M 2201
Ductility (15 °C, 5 cm/min) (cm)	150	KS M 2254
Softening (°C)	47.5	KS M 2250
Flash Point (°C)	354	KS M 2010
Density (15 °C) (kg/m^3^)	1.041	KS M 2256

KS Standards available in reference [41].

**Table 2:** ESGFIBER Properties.

Property	Test Value	Standards
Cross section area (mm^2^)	0.85	-
Length (mm)	12~16	-
Thickness (mm)	1.04	-
Tensile Strength (MPa)	3750	ASTM D2101
Elongation at break (%)	4.9	ASTM D2101
Elastic Modulus (GPa)	81	ASTM D2101
Density (g/cm^3^)	2.66	ASTM C693
Reflective Index	1.567	ASTM C1648
Conductivity (watts/m.K)	1.22	ASTM C177
Coefficient of Thermal expansion (10^6^/°C)	6.0	ASTM D689

**Table 3:** Volumetric properties of asphalt mixtures.

Properties	Control Mix (0%)	Mix 1 (0.2%)	Mix 2 (0.3%)	Mix 3 (0.4%)	Mix 4 (0.5%)
OAC (%)	5	4.8	4.75	4.7	4.65
G_mb_ (g/cm^3^)	2.404	2.404	2.405	2.406	2.408
G_mm_ (g/cm^3^)	2.504	2.506	2.507	2.507	2.508
VMA (%)	15.58	15.22	15.10	14.60	14.84
VFA (%)	74.53	73.29	73.57	72.38	72.91
Air Voids @4.5%	5.17	4.83	4.79	4.73	4.43
Air Voids @5%	4.01	3.63	3.27	3.01	2.99
Air Voids @5.5%	2.26	2.18	1.76	1.63	1.58
Stability @4.5%	12,582.67	12,644.68	12,818.6	12,847.47	12,892.5
Stability @5%	11,169.6	12,511.98	12,636.67	12,711.82	12,678.31
Stability @5.5%	10,714.17	11,277.23	11,168.9	11,480.1	11,613.58

## Data Availability

Not applicable.
